# Association between 20% Albumin Use and Acute Kidney Injury in Major Abdominal Surgery with Transfusion

**DOI:** 10.3390/ijms24032333

**Published:** 2023-01-25

**Authors:** Hye Jin Kim, Hyun Joo Kim, Jin Ha Park, Hye Jung Shin, Sung Kyung Yu, Yun Ho Roh, Soo Yeon Jeon, So Yeon Kim

**Affiliations:** 1 Department of Anesthesiology and Pain Medicine, Anesthesia and Pain Research Institute, Yonsei University College of Medicine, 50-1 Yonsei-ro, Seodaemun-gu, Seoul 03722, Republic of Korea; 2 Biostatistics Collaboration Unit, Department of Biomedical Systems Informatics, Yonsei University College of Medicine, 50-1 Yonsei-ro, Seodaemun-gu, Seoul 03722, Republic of Korea; 3 Division of Healthcare Big Data, Yonsei University Health System, 50-1 Yonsei-ro, Seodaemun-gu, Seoul 03722, Republic of Korea

**Keywords:** abdominal surgery, acute kidney injury, albumin, blood transfusion, hospitalization, mortality

## Abstract

Red blood cell (RBC) transfusion and albumin administration can affect kidney function. We aimed to evaluate the association between intraoperative 20% albumin administration and acute kidney injury (AKI), along with the duration of hospitalization and 30-day mortality in patients undergoing major abdominal surgery with RBC transfusion. This retrospective study included 2408 patients who received transfusions during major abdominal surgery. Patients were categorized into albumin (*n* = 842) or no-albumin (*n* = 1566) groups. We applied inverse probability of treatment weighting (IPTW), propensity score (PS) matching (PSM), and PS covariate adjustment to assess the effect of albumin administration on the outcomes. In the unadjusted cohort, albumin administration was significantly associated with increased risk of AKI, prolonged hospitalization, and higher 30-day mortality. However, there was no significant association between albumin administration and AKI after adjustment (OR 1.26, 95% CI 0.90–1.76 for the IPTW; OR 1.03, 95% CI 0.72–1.48 for the PSM; and OR 1.04, 95% CI 0.76–1.43 for the PS covariate adjustment methods). While albumin exposure remained associated with prolonged hospitalization after adjustment, it did not affect 30-day mortality. Our findings suggest that hyper-oncotic albumin can be safely administered to patients who are at risk of developing AKI due to RBC transfusion.

## 1. Introduction

Hypoalbuminemia occurs frequently during major abdominal surgery. A previous study reported a 43% decrease in plasma albumin levels during the initial steps of major abdominal surgery [1], possibly due to increased capillary permeability to albumin from surgical trauma [2,3]. Moreover, bleeding followed by fluid resuscitation and transfusion can induce hypoalbuminemia in major abdominal surgery [2,4]. Human albumin solutions, including hypo-oncotic (4%), iso-oncotic (5%), and hyper-oncotic (20–25%) formulations, are used to counteract hypoalbuminemia or for volume replacement [5,6,7]. The use of hyper-oncotic albumin for resuscitation may have an advantage over iso-/hypo-oncotic albumin formulations by reducing fluid resuscitation requirements and cumulative fluid balance [6,8].

The incidence of acute kidney injury (AKI) after major abdominal surgery ranges from 3 to 35%, and AKI is closely associated with prolonged hospitalization and increased morbidity and mortality [9,10,11,12]. Numerous studies have reported the effects of exogenous hyper-oncotic albumin on AKI in various clinical settings; however, the results have been inconsistent, with some studies reporting harmful, beneficial, or no effects [5,7,13,14,15,16,17,18,19]. Albumin is commonly administered in patients receiving red blood cell (RBC) transfusion during major abdominal surgery to correct hypoalbuminemia and volume depletion. However, little is known regarding the effects of hyper-oncotic albumin on kidney function in patients receiving RBC transfusions. Intraoperative RBC transfusion is a known predictor of AKI following major abdominal surgery [9,10,11,12,20], and a greater degree of caution is required to prevent AKI in patients receiving transfusions. Therefore, this retrospective study aimed to evaluate the association between intraoperative 20% albumin administration and postoperative AKI in patients undergoing major abdominal surgery with RBC transfusion. Additionally, clinical outcomes, such as the duration of hospitalization and 30-day mortality, were evaluated.

## 2. Results

### 2.1. Study Cohort and Patient Characteristics

**Figure 1:**
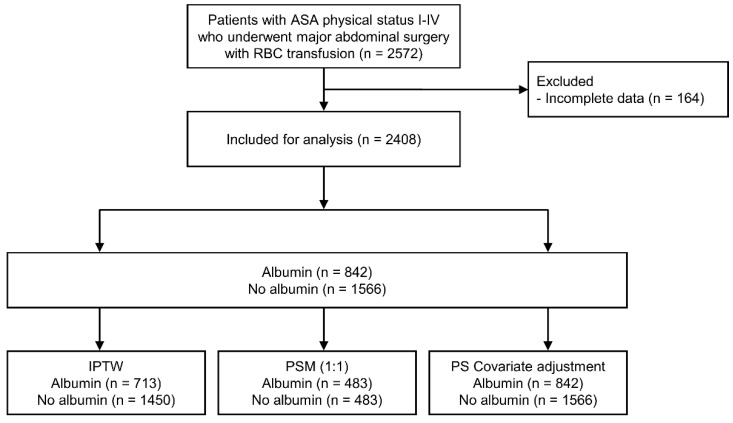
Flow chart of patient selection. ASA, American Society of Anesthesiologists; RBC, red blood cell; IPTW, inverse probability of treatment weighting; PSM, propensity score matching; PS, propensity score.

**Figure 2:**
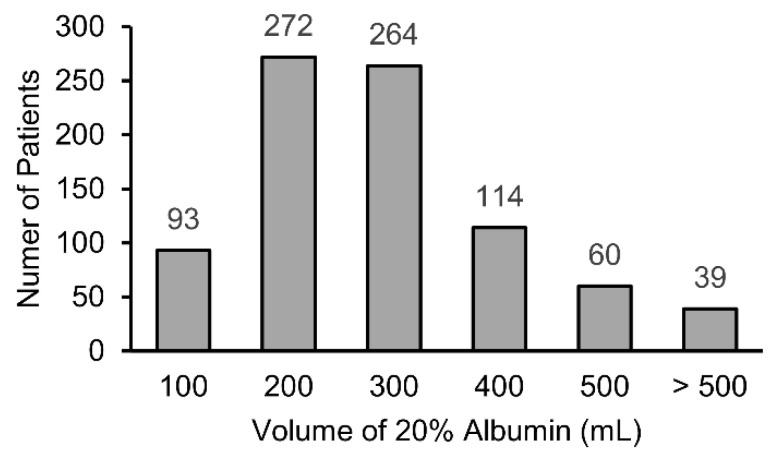
The volume of 20% albumin solution administered for each patient.

**Figure 3:**
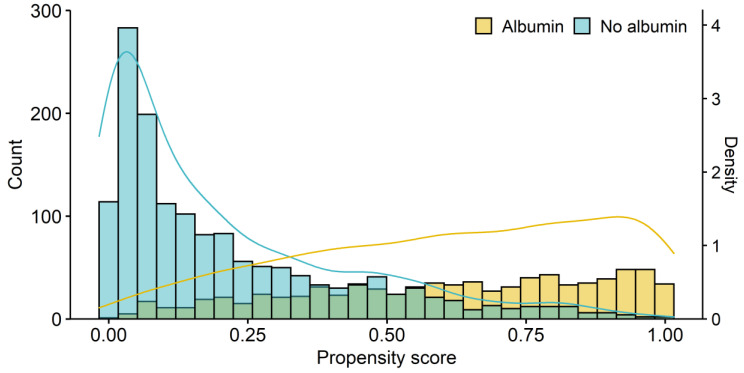
Distribution of propensity scores for patients in the albumin and no-albumin groups.

**Figure 4:**
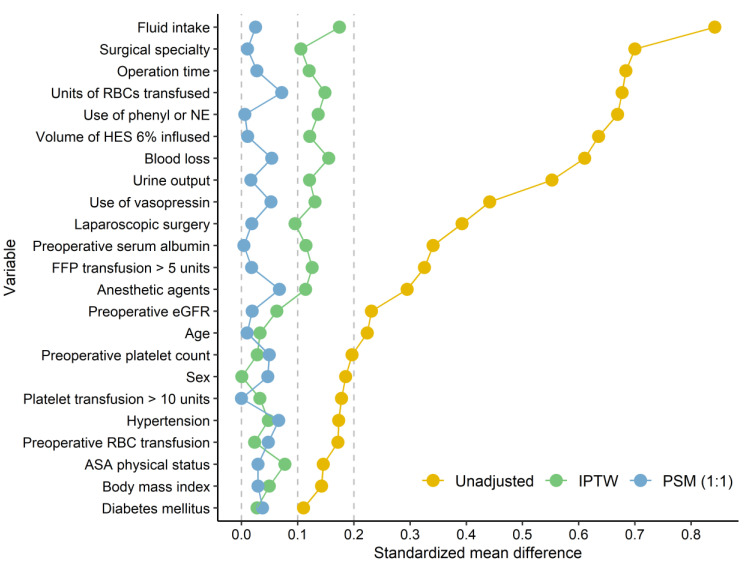
SMD of the preoperative and intraoperative covariates in the unadjusted and adjusted cohorts. SMD, standardized mean difference; IPTW, inverse probability of treatment weighting; PSM, propensity score matching; RBC, red blood cell; phenyl, phenylephrine; NE, norepinephrine; HES, hydroxyethyl starch; FFP, fresh frozen plasma; eGFR, estimated glomerular filtration rate; ASA, American Society of Anesthesiologists.

Among the 2572 patients initially screened, 164 with incomplete data were excluded. Ultimately, 2408 patients were included in the analysis, 842 of whom received albumin during surgery (Figure 1). The distribution of the volume of albumin administered is shown in Figure 2. In most cases, either 200 mL or 300 mL of 20% albumin was administered. The PS distributions in the albumin and no-albumin groups are shown in Figure 3. Baseline demographic, clinical, and intraoperative characteristics in the unadjusted and adjusted (IPTW and PSM) cohorts are shown in Table 1 and Table 2, respectively. For the preoperative and intraoperative covariates with an SMD > 0.1 in the unadjusted analysis, a comparison of the extent of the imbalance between the unadjusted and adjusted (IPTW and PSM) cohorts is depicted in Figure 4.

### 2.2. Primary and Secondary Outcomes

**Figure 5:**
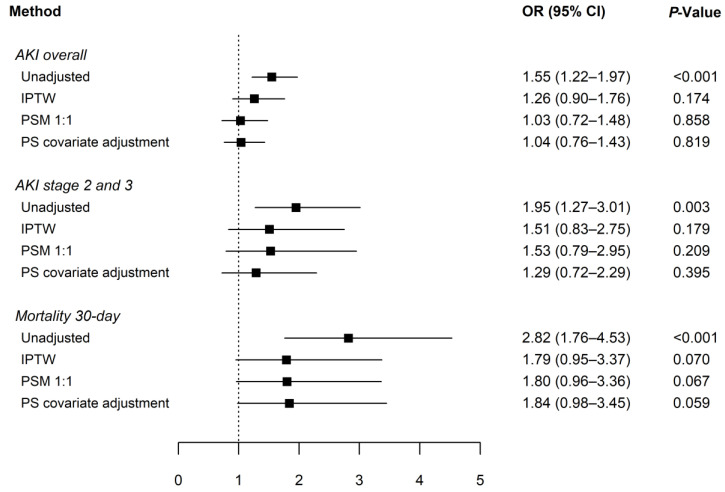
Forest plot showing the association of intraoperative 20% albumin administration with the occurrence of AKI and 30-day mortality. Solid squares represent the odds ratio (OR), and the horizontal line represents the 95% confidence interval (CI). AKI, acute kidney injury; IPTW, inverse probability of treatment weighting; PSM, propensity score matching; PS, propensity score.

In the unadjusted cohort, AKI occurred in 13.3% (321 of 2408) of patients, and the incidence of AKI was higher in the albumin group than in the no-albumin group (16.7% versus 11.5%, respectively; *p* < 0.001). The forest plot depicts the OR of albumin administration for the occurrence of AKI and 30-day mortality (Figure 5). In all three adjusted analyses, there was no significant association between albumin administration and overall AKI (OR 1.26, 95% CI: 0.90–1.76 for the IPTW; OR 1.03, 95% CI: 0.72–1.48 for the PSM; and OR 1.04, 95% CI: 0.76–1.43 for the PS covariate adjustment methods); there was also no association with stage 2 and 3 AKI (OR 1.51, 95% CI: 0.83–2.75 for the IPTW; OR 1.53, 95% CI: 0.79–2.95 for the PSM; and OR 1.29, 95% CI: 0.72–2.29 for the PS covariate adjustment methods), although significant associations existed in the unadjusted cohorts. Similarly, albumin administration exhibited no association with 30-day mortality after adjustment with any of the three PS methods, despite significant associations being observed before adjustment. However, a statistically significant association remained between albumin administration and prolonged postoperative hospitalization, even after adjustment (β = 4.01, 95% CI: 1.93–6.09 for the IPTW; β = 3.08, 95% CI: 1.32–5.04 for the PSM; and β = 3.11, 95% CI: 1.53–4.68 for the PS covariate adjustment methods) (Table 3).

## 3. Discussion

This study is the first to assess the relationship between intraoperative 20% albumin administration and AKI occurrence after major abdominal surgery with RBC transfusion. There was no association of albumin administration with increased AKI or 30-day mortality after adjusting for confounders using different PS methods, despite an association with prolonged hospitalization.

Under physiological conditions, approximately 40% and 60% of albumin is located in intravascular and extravascular compartments, respectively; however, critical illness and major surgical stress alter this distribution through increased capillary leakage [2,3]. A previous study demonstrated a 43% decrease in plasma albumin levels during the first portion of major abdominal surgery, which progressed until 1 h after surgery and remained stable thereafter for up to 72 h [1]. Therefore, hypoalbuminemia is frequently encountered during abdominal surgery, especially in patients receiving RBC transfusion due to transcapillary leakage of albumin, blood loss, and dilution from fluid resuscitation [4].

Human albumin solutions, including hypo-/iso-oncotic (4–5%) or hyper-oncotic (20–25%) formulations, are administered to correct hypoalbuminemia and expand intravascular volumes [5,6,7]. Numerous studies have investigated associations between albumin administration and patient outcomes, including AKI and mortality, under various conditions; however, the results have been inconsistent, with some showing harmful, beneficial, or no effects [5,7,13,14,15,16,17,18,19]. In patients undergoing on-pump cardiac surgery, 5% or 25% albumin administration was associated with dose-dependent increases in AKI risk [13]. Contrarily, 20% albumin administration immediately before surgery reduced AKI risk after off-pump coronary artery bypass surgery [16]. Meanwhile, early hyper-oncotic albumin exposure during the first 48 h of postoperative shock was associated with greater AKI risk, particularly among patients undergoing cardiac surgery [15].

However, the use of 5% or 25% albumin in patients experiencing bleeding after cardiac surgery during the early perioperative period was not associated with the occurrence of AKI or mortality [19]. In addition, in a large-scale meta-analysis that evaluated hypo-, iso-, and hyper-oncotic albumin administration under several clinical conditions, no effect of albumin on mortality was detected [5]. Therefore, the effects of albumin on patient outcomes can differ depending on the medical or surgical conditions. A meta-analysis showed a significant benefit in terms of morbidity in patients with ascites, a trend toward reduced morbidity in patients with burns or hypoalbuminemia, and no beneficial effect in those undergoing surgery or experiencing traumatic injury [7].

Another recent meta-analysis concluded that hyper-oncotic albumin should be used more routinely to avoid the potential risks associated with a positive fluid balance that are encountered with hypo-oncotic solutions [6]. However, the efficacy of hyper-oncotic albumin administration during major abdominal surgery remains unclear. In previous studies using 20% albumin in major abdominal surgery, there were no differences in kidney function and other postoperative complications between the albumin and no-albumin groups [21,22]. However, in those two studies, albumin was administered during the early postoperative period rather than intraoperatively [21,22]. Moreover, the number of patients may have been insufficient to identify the renal effects of albumin, as none of the 127 patients enrolled in the study developed postoperative kidney dysfunction [22]. Hence, our study is valuable in that it is the first study conducted with a large number of patients to identify an association between intraoperative albumin administration and AKI after major abdominal surgery. Moreover, we focused on patients receiving intraoperative RBC transfusion, as this is a known risk factor for AKI [9,10,11,12,20].

Before adjustment, the patients who received 20% albumin exhibited a greater risk of AKI than those who did not. However, albumin administration appeared not to be associated with AKI after adjustment for confounders using various PS methods. Even when considering severe AKI (stages 2 and 3), intraoperative hyper-oncotic albumin exposure did not increase the risk of AKI after adjusting for the confounders. Similarly, the significant association between albumin administration and higher 30-day mortality disappeared after the adjustments. The differences in the results between the unadjusted and adjusted cohorts may be attributable to the markedly different characteristics between the albumin and no-albumin groups before adjustment, as the SMD was > 0.1 for most of the preoperative and intraoperative variables. Therefore, exposure to 20% albumin in patients receiving RBC transfusion appears to be safe in terms of kidney function after major abdominal surgery.

Although albumin administration was not associated with increased AKI risk, it was significantly associated with prolonged postoperative hospitalization (approximately 3–4 days), even after adjusting for confounders. Perioperative RBC transfusion and albumin administration have been reported to be independent predictors of postoperative infection after colorectal surgery [23]. Additionally, albumin supplementation within 30 days after spinal surgery increased surgical-site infection rates [24]. Therefore, the possible cause of the prolonged hospitalization in patients receiving albumin might have been related to postoperative infection, which may result in longer hospitalization after major abdominal surgery [25,26,27]. Nevertheless, prolonged hospitalization in patients receiving albumin did not result in higher 30-day mortality rates in our study.

Our study has some limitations. First, the data were collected retrospectively at a single center, increasing the possibility of bias and the influence of confounding factors. However, the incidence of AKI was comparable to that reported by previous studies (13–22%) using KDIGO criteria in major abdominal surgery [10,11,28], and we adjusted for confounding factors using different PS methods. Nevertheless, it is possible that the outcomes were influenced by unknown and unadjusted confounding factors. This calls for randomized controlled trials with sufficient sample size. Second, albumin was administered at the discretion of the attending anesthesiologists, and the reasons for albumin administration, such as hypoalbuminemia and volume expansion, were unknown. Although we adjusted for preoperative serum albumin levels, a prospective study with a defined indication for albumin administration may be needed for more controlled results. Finally, we could not evaluate intraoperative hemodynamic instability, which may affect AKI occurrence [29]. Although we adjusted for several variables (fluids/blood products administered, urine output, blood loss, anesthetic agents, operation time, vasopressor use, and type of surgery), data on intraoperative blood pressure and heart rate, representing intraoperative hemodynamics, were unavailable.

## 4. Materials and Methods

### 4.1. Study Design and Ethical Approval

This single-center historical cohort study was performed following approval from the Institutional Review Board and Hospital Research Ethics Committee of Severance Hospital, Yonsei University Health System, Seoul, Korea (IRB number: 4-2021-1503; approval date: 17 December 2021). The requirement for informed consent was waived due to the retrospective nature of the study. This study followed the guidelines of the Declaration of Helsinki (1964) and its later amendments (2013).

### 4.2. Selection and Description of Participants

The study included patients aged > 18 years who underwent major abdominal surgery (elective or emergency) between March 2014 and January 2020, had an American Society of Anesthesiologists (ASA) physical status of I–IV, and received at least 1 unit of RBCs transfused intraoperatively. Major abdominal surgery was defined as surgery involving the abdominal and/or retroperitoneal compartment, with the removal of all or part of an organ, lasting ≥ 1 h. We excluded patients with incomplete data. Patients were divided into two groups according to whether albumin was administered. All patients in the albumin group received 20% human albumin (Green Cross Co., Yong-In, Republic of Korea).

### 4.3. Data Collection

The following data were retrospectively collected from patients’ electronic medical records: age, sex, body mass index, ASA physical status, the presence of comorbidities (hypertension, diabetes mellitus, cerebrovascular disease, asthma, coronary artery disease, valvular heart disease, atrial fibrillation, and chronic kidney disease), and the history of preoperative RBC transfusion (within 2 days before surgery). Preoperative laboratory data collected included the platelet count, serum albumin and hematocrit levels, and estimated glomerular filtration rate (eGFR). The eGFR was calculated from serum creatinine (SCr) levels using the Chronic Kidney Disease Epidemiology Collaboration equation [30], and chronic kidney disease was defined as an eGFR < 60 mL·min^−1^·1.73 m^−2^. The following intraoperative data were recorded: operation time, anesthetic agents administered (desflurane versus sevoflurane versus propofol for total intravenous anesthesia), vasopressor use (phenylephrine/norepinephrine and vasopressin), total fluid intake volume, infusion volume of hydroxyethyl starch (6% 130/0.4) solution (Volulyte, Fresenius Kabi, Bad Homberg, Germany), urine output, blood loss, units transfused (RBCs, fresh frozen plasma, and platelet concentrates), type of surgery (laparoscopic, open, cancer, elective, or emergency surgery), and the surgical specialty (upper gastrointestinal, hepatobiliary, colorectal, gynecological). Furthermore, the SCr levels within the first 7 postoperative days, duration of postoperative hospitalization, and 30-day mortality rate were obtained.

### 4.4. Study Endpoints

The primary endpoint was the occurrence of AKI according to the Kidney Disease: Improving Global Outcomes (KDIGO) criteria (increase in SCr level of ≥0.3 mg·dL^−1^ within 48 h or an increase in SCr level to ≥1.5-times baseline within 7 days following surgery). AKI was further categorized into the following three stages: stage 1, an increase in SCr level 1.5–1.9-times baseline or by ≥0.3 mg·dL^−1^; stage 2, an increase in SCr level 2.0–2.9-times baseline; stage 3, an increase in SCr level at least 3.0-times baseline or to ≥4.0 mg·dL^−1^, or the initiation of renal replacement therapy [31]. The overall occurrence of AKI and its occurrence based on severity (stages 2 and 3) were evaluated. The secondary endpoints were the duration of postoperative hospitalization and 30-day mortality.

### 4.5. Statistical Analysis

Continuous variables are described as median values (interquartile range), whereas categorical variables are expressed as numbers (percentages). We analyzed the effect of intraoperative 20% albumin administration on the study outcomes using logistic regression and linear regression. This was achieved using the following three methods of adjusting for the effects of potential confounding variables to generate unbiased estimates: inverse probability of treatment weighting (IPTW), propensity score (PS) matching (PSM), and PS covariate adjustment. The demographic, clinical, and intraoperative variables exhibiting standardized mean differences (SMDs) of >0.1 between the albumin and no-albumin groups were chosen as the adjustment variables. The PS was calculated from the logistic regression model, with the group as the dependent variable and the adjustment factors as independent variables.

For the analysis involving IPTW, we used stabilized trimmed weights at the 99th percentile to minimize extreme weight influences; these were modifications to the usual weights of 1/PS for the albumin group and 1/(1-PS) for the no-albumin group [32]. For the PSM method, we performed 1:1 nearest-neighbor matching with a 0.1 caliper. The PS covariate adjustment method used the PS as a covariate in the regression model for estimating the effect of albumin. The balance of the demographic, clinical, and intraoperative variables was evaluated based on the SMDs in the unadjusted and adjusted (IPTW and PSM) cohorts. The unadjusted and adjusted odds ratios (ORs) of albumin administration, along with the 95% confidence intervals (CIs), were determined for the binary outcomes. OR values were calculated via the logistic regression model with robust variance estimates. Unadjusted and adjusted β values for albumin administration were calculated for the continuous outcome variables, along with their 95% CIs. β values were estimated as follows: by the linear regression model for the unadjusted and PS covariate adjustment methods; by the linear regression model with robust variance estimates for the IPTW method; and by the linear mixed model for the PSM method. *p* < 0.05 was considered statistically significant, and all tests were two-tailed. We used SAS version 9.4 (SAS Institute Inc., Cary, NC, USA) software for all statistical analyses.

## 5. Conclusions

Intraoperative 20% albumin administration was not associated with increased AKI risk or 30-day mortality after adjusting for confounders in patients undergoing major abdominal surgery with RBC transfusion, although it was related to prolonged hospitalization. Therefore, hyper-oncotic albumin can be safely administered to patients who are at risk of developing AKI due to RBC transfusion. Further randomized controlled trials assessing various postoperative complications should be performed to assess the safety of intraoperative hyper-oncotic albumin in major abdominal surgery.

## Figures and Tables

**Table 1:** Demographic and clinical characteristics in the unadjusted and adjusted cohorts.

Variables	Unadjusted			Adjusted Using IPTW			Adjusted Using PSM		
	Albumin (*n* = 842)	No Albumin (*n* = 1566)	SMD	ALBUMIN (*n* = 713)	No Albumin (*n* = 1450)	SMD	Albumin (*n* = 483)	No Albumin (*n* = 483)	SMD
Age (years)	59 [50–69]	64 [52–73]	0.224	60 [51–71]	62 [50–72]	0.033	60 [50–71]	61 [50–72]	0.010
Male sex	294 (34.9%)	688 (43.9%)	0.185	298 (41.8%)	607 (41.9%)	0.001	183 (37.9%)	194 (40.2%)	0.047
Body mass index (kg·m^−2^)	22.5 [20.2–24.7]	23.0 [20.8–25.2]	0.143	22.6 [20.5–25.1]	22.8 [20.5–25.2]	0.049	22.5 [20.3–24.8]	22.4 [20.0–24.7]	0.029
ASA physical status			0.145			0.077			0.030
I	30 (3.6%)	96 (6.1%)		28 (3.9%)	79 (5.4%)		21 (4.4%)	22 (4.6%)	
II	315 (37.4%)	609 (38.9%)		287 (40.2%)	558 (38.5%)		191 (39.5%)	188 (38.9%)	
III	424 (50.4%)	759 (48.5%)		345 (48.4%)	708 (48.8%)		229 (47.4%)	234 (48.5%)	
IV	73 (8.7%)	102 (6.5%)		54 (7.6%)	106 (7.3%)		42 (8.7%)	39 (8.1%)	
Comorbidities									
Hypertension	392 (46.6%)	864 (55.2%)	0.173	344 (48.2%)	733 (50.6%)	0.048	226 (46.8%)	242 (50.1%)	0.066
Diabetes mellitus	224 (26.6%)	495 (31.6%)	0.110	197 (27.6%)	418 (28.9%)	0.028	126 (26.1%)	134 (27.7%)	0.037
Cerebrovascular disease	49 (5.8%)	116 (7.4%)	0.064	42 (5.9%)	96 (6.6%)	0.031	32 (6.6%)	29 (6.0%)	0.026
Asthma	18 (2.1%)	43 (2.8%)	0.039	12 (1.7%)	41 (2.8%)	0.072	11 (2.3%)	16 (3.3%)	0.063
Coronary artery disease	23 (2.7%)	72 (4.6%)	0.099	22 (3.1%)	68 (4.7%)	0.082	16 (3.3%)	21 (4.4%)	0.054
Valvular heart disease	16 (1.9%)	36 (2.3%)	0.028	11 (1.5%)	39 (2.7%)	0.082	9 (1.9%)	16 (3.3%)	0.091
Atrial fibrillation	18 (2.1%)	44 (2.8%)	0.043	23 (3.3%)	35 (2.4%)	0.051	9 (1.9%)	10 (2.1%)	0.015
Chronic kidney disease	11 (1.3%)	39 (2.5%)	0.087	11 (1.6%)	31 (2.1%)	0.039	8 (1.7%)	10 (2.1%)	0.031
Preoperative RBC transfusion	77 (9.1%)	75 (4.8%)	0.172	47 (6.6%)	87 (6.0%)	0.023	38 (7.9%)	32 (6.6%)	0.048
Preoperative laboratory values									
Hematocrit (%)	32.9 [29.3–37.0]	33.0 [28.9–37.4]	0.020	33.0 [29.1–37.5]	32.9 [28.8–37.3]	0.055	33.0 [29.3–37.6]	32.7 [28.6–36.9]	0.125
Platelet count (×10^3^·μL^−1^)	275 [201–369]	252 [190–323]	0.197	260 [184–344]	256 [191–332]	0.028	275 [200–359]	265 [201–343]	0.050
Serum albumin (g·dL^−1^)	3.6 [2.9–4.3]	4.0 [3.4–4.3]	0.341	3.8 [3.0–4.4]	3.9 [3.3–4.3]	0.115	3.7 [3.0–4.4]	3.7 [3.1–4.2]	0.004
eGFR (mL·min^−1^·1.73 m^−2^)	98 [85–108]	92 [78–104]	0.231	97 [84–107]	94 [80–106]	0.063	97 [83–108]	97 [83–109]	0.019

Values are expressed as the median (interquartile range) or number of patients (percentage). IPTW, inverse probability of treatment weighting; PSM, propensity score matching; SMD, standardized mean difference; ASA, American Society of Anesthesiologists; RBC, red blood cell; eGFR, estimated glomerular filtration rate.

**Table 2:** Intraoperative characteristics in the unadjusted and adjusted cohorts.

Variables	Unadjusted			Adjusted Using IPTW			Adjusted Using PSM		
	Albumin (n = 842)	No Albumin (n = 1566)	SMD	Albumin (n = 713)	No Albumin (n = 1450)	SMD	Albumin (n = 483)	No Albumin (n = 483)	SMD
Operation time (min)	411 [264–588]	275 [191–396]	0.684	347 [207–505]	311 [214–467]	0.121	356 [216–502]	344 [235–494]	0.027
Anesthetic agents			0.295			0.114			0.068
Desflurane	414 (49.2%)	952 (60.8%)		380 (53.3%)	843 (58.1%)		250 (51.8%)	266 (55.1%)	
Sevoflurane	418 (49.6%)	565 (36.1%)		321 (44.9%)	573 (39.5%)		227 (47.0%)	212 (43.9%)	
Propofol for TIVA	10 (1.2%)	49 (3.1%)		13 (1.8%)	35 (2.4%)		6 (1.2%)	5 (1.0%)	
Vasopressor use									
Phenyl or NE	771 (91.6%)	1026 (65.5%)	0.669	569 (79.8%)	1074 (74.0)	0.137	419 (86.8%)	418 (86.5%)	0.006
Vasopressin	159 (18.9%)	77 (4.9%)	0.442	86 (12.1%)	118 (8.1%)	0.131	58 (12.0%)	50 (10.4%)	0.053
Fluid intake (mL)	5250 [3513–7300]	3000 [2085–4388]	0.842	4200 [2500–5900]	3550 [2450–5200]	0.174	4300 [2700–5900]	3950 [2750–5600]	0.025
HES 6% infused (mL)	1000 [1000–1500]	1000 [500–1000]	0.636	1000 [500–1000]	1000 [500–1000]	0.121	1000 [500–1000]	1000 [500–1000]	0.011
Urine output (mL)	745 [390–1360]	455 [240–780]	0.552	590 [279–1075]	515 [260–960]	0.121	600 [308–1053]	580 [310–1053]	0.017
Blood loss (mL)	1250 [650–2250]	650 [300–1100]	0.611	900 [400–1700]	800 [400–1400]	0.155	900 [500–1695]	850 [500–1500]	0.054
Transfusion									
RBCs (U)	3 [2–5]	1 [1–2]	0.677	2 [1–3]	2 [1–2]	0.149	2 [1–3]	2 [1–3]	0.072
FFP > 5 U	51 (6.1%)	6 (0.4%)	0.326	20 (2.8%)	15 (1.0%)	0.126	7 (1.5%)	6 (1.2%)	0.018
Platelets > 10 U	21 (2.5%)	6 (0.4%)	0.178	9 (1.3%)	14 (0.9%)	0.033	4 (0.8%)	4 (0.8%)	<0.001
Laparoscopic surgery	84 (10.0%)	384 (24.5%)	0.392	106 (14.9%)	267 (18.4%)	0.096	66 (13.7%)	63 (13.0%)	0.018
Cancer surgery	672 (79.8%)	1252 (80.0%)	0.004	572 (80.2%)	1142 (78.7%)	0.037	376 (77.9%)	371 (76.8%)	0.025
Emergency surgery	147 (17.5%)	232 (14.8%)	0.072	130 (18.2%)	242 (16.7%)	0.040	95 (19.7%)	92 (19.1%)	0.016
Surgical specialty			0.700			0.106			0.011
Upper GI	66 (7.8%)	304 (19.4%)		107 (15.0%)	229 (15.8%)		57 (11.8%)	58 (12.0%)	
Hepatobiliary	42 (5.0%)	349 (22.3%)		95 (13.3%)	243 (16.8%)		36 (7.5%)	35 (7.3%)	
Colorectal	425 (50.5%)	564 (36.0%)		305 (42.7%)	580 (40.0%)		233 (48.2%)	234 (48.5%)	
Gynecological	309 (36.7%)	349 (22.3%)		207 (29.0%)	397 (27.4%)		157 (32.5%)	156 (32.3%)	

Values are expressed as the median (interquartile range) or number of patients (proportion). IPTW, inverse probability of treatment weighting; PSM, propensity score matching; SMD, standardized mean difference; TIVA, total intravenous anesthesia; Phenyl, phenylephrine; NE, norepinephrine; HES, hydroxyethyl starch; RBCs, red blood cells; FFP, fresh frozen plasma; U, units; GI, gastrointestinal.

**Table 3:** Association of intraoperative 20% albumin administration with the duration of postoperative hospitalization (days) before and after adjustment.

Analysis Method	β (95% CI)	*p*-Value
Unadjusted	6.15 (4.96 to 7.35)	<0.001
Adjustment with IPTW	4.01 (1.93 to 6.09)	<0.001
Adjustment with PSM	3.18 (1.32 to 5.04)	<0.001
PS covariate adjustment	3.11 (1.53 to 4.68)	<0.001

CI, confidence interval; IPTW, inverse probability of treatment weighting; PSM, propensity score matching; PS, propensity score. The adjustment methods (IPTW, PSM, and PS covariate adjustment) used the following variables as covariates: age, sex, body mass index, American Society of Anesthesiologists physical status, hypertension, diabetes mellitus, preoperative red blood cell transfusion, preoperative platelet count, preoperative serum albumin level, preoperative estimated glomerular filtration rate, operation time, anesthetic agents, use of phenylephrine /norepinephrine, use of vasopressin, total fluid intake volume, infusion volume of hydroxyethyl starch 6%, urine output, blood loss, units of red blood cells transfused, fresh frozen plasma transfusion > 5 units, platelet transfusion > 10 units, laparoscopic surgery, and surgical specialty.

## Data Availability

The data presented in this study are available on request from the corresponding author. The data are not publicly available due to privacy issue.
